# Analysis of the Introduction in Clinical Practice of New Oral Anticoagulants in Local Health Agency BT: Translation of the Clinical Trial Data to a Local Health Care Area

**DOI:** 10.3390/pharmaceutics13020252

**Published:** 2021-02-11

**Authors:** Gianluca Grimaldi, Domenica Ancona, Domenico Tricarico, Paolo Stella, Cataldo Procacci, Antonio Germinario, Vito Bavaro, Vito Montanaro, Alessandro Delle Donne

**Affiliations:** 1 Local Health Agency BT, via Fornaci 201, 76123 Andria, Italy; gianluca.grimaldi@aslbat.it (G.G.); aldo.procacci@aslbat.it (C.P.); antonio.germinario@aslbat.it (A.G.); avvocato.aledelledonne@gmail.com (A.D.D.); 2 Department of Pharmacy-Pharmaceutical Sciences, University Aldo Moro, via Orabona 4, 70125 Bari, Italy; domenico.tricarico@uniba.it; 3 Department for the Promotion of Health, Social Welfare and Sport for All, via Gentile 52, 70126 Bari, Italy; p.stella@regione.puglia.it (P.S.); v.bavaro@regione.puglia.it (V.B.); vito.montanaro@regione.puglia.it (V.M.)

**Keywords:** NOAC, VKA, real-world data, direct health costs

## Abstract

The commercial release of the New Oral Anticoagulants (NOACs) has been the most significant change in anticoagulant therapy in recent years. The work aimed to evaluate the economic and health impact for the Local Health Agency Barletta-Andria-Trani (BT). Through the Regional Information System data about naïve patients on NOAC treatment and patients on anti-vitamin-k (VKA), treatments were extrapolated. We assessed therapeutic continuity, pharmaceutical expenditure, hospitalizations, and deaths in 2017 and 2018. Therapeutic continuity was similar in the two groups. The number and the average cost of hospitalizations for a patient treated with VKAs were almost constant, while those of patients treated with NOACs decreased. The treatment of adult-aged naïve patients with NOACs, compared to VKAs therapy, involves an increase in expenditure of about 100€ for a patient, but the reduced hospitalizations could generate, in the long term, saving for the Health System. Clinical data, according to the Real-World Data, confirmed the safety and effectiveness of these drugs. However, attention to the special population is necessary to improve the safety and effectiveness of NOACs. Innovative formulations for pediatric patients are being developed. The challenge for Health Systems is the appropriate use of available resources through health interventions with transversal competences.

## 1. Introduction

Atrial fibrillation (AF) is the most common cardiac arrhythmia in developed countries and is independently associated with an increased risk of mortality and high morbidity as well as frequent hospitalizations and worsening of life quality [1].

Venous thromboembolism (VTE) is, likewise, one of the largest clinical and economic burdens worldwide. Anticoagulant treatment is necessary to immediately reduce short-term and long-term VTE-related morbidity and mortality. Also, late complications, such as post-thrombotic syndrome (PTS), are common and have a significant economic-health burden [1]. In past years, low-molecular-weight heparins (LMWH), fondaparinux, and vitamin K antagonists (VKAs) have been the standard of anticoagulant therapy, but these therapeutic options have clinically relevant disadvantages and limitations.

The commercial release of the New Oral Anticoagulants (NOACs) has been the most significant change in anticoagulant therapy in recent years. They show a rapid start and offset of action, lower interactions with food and drugs, predictable anticoagulant effect, and absence of the need for routine monitoring of clotting, as well as a better safety profile [2].

All NOACs were compared in head-to-head studies with warfarin and/or enoxaparin to assess both effectiveness on stroke, systemic embolism (SE), and VTE, as well as the safety of treatments. Although similar in some aspects, clinical studies are not directly comparable. When compared to warfarin, all NOACs show a favorable safety and efficacy profile for stroke and SE prevention in patients with non-valve atrial fibrillation (NVAF). However, statistical superiority has not been demonstrated by all of them and for all effectiveness and security endpoints [3,4]. The results of the analysis of the bleeding observed in the different body districts, executed in the trials, show important differences between both the different NOACs and warfarin [4,5,6].

In the treatment of deep venous thrombosis (DVT) and pulmonary embolism (PE), all NOACs were compared with conventional enoxaparin/warfarin therapy, while in the prevention of DVT and PE they were compared vs. placebo. In the acute treatment of DVT and PE, all NOACs are superimposed on conventional therapy in reducing recurrences and related death. In preventive treatments, as expected, NOACs are better than placebo in preventing recurrences and related mortality. The evaluation of the safety data provides not homogeneous feedback that more closely reflects the pharmacological differences of the individual molecules and the different choices about their administration [7]. Following the commercial release, the scientific evidence increased exponentially, and retrospective and observational studies were able to assess outcomes such as intracranial hemorrhage and gastrointestinal (GI) hemorrhage in heterogeneous populations of patients for each NOAC. Pharmacological properties and some Real-World Data are reported in Supplementary Material 1 [8,9,10,11,12,13,14,15,16,17,18,19,20,21,22,23,24,25,26,27,28,29,30,31,32,33,34,35,36].

Furthermore, pharmacoeconomics studies analyzed the cost effectiveness of these drugs. 

Amin et al. analyzed medical costs that may be avoided when a NOAC is used instead of warfarin in the United States. Medical cost differences were estimated based on clinical trials’ results. They considered a hypothetical population of 1 million people and results were projected in the years 2015–2018.

They found that in 2014, for NVAF patients treated with a NOAC (dabigatran, rivaroxaban, apixaban, and edoxaban), medical costs were reduced by $3.0 million, $2.1 million, $7.3 million, and $5.0 million, respectively. Likewise, for acute VTE patients, medical costs were reduced by $0.7 million, $2.2 million, $4.1 million, and $1.6 million. For the combined NVAF and acute VTE patient population medical costs were projected to be reduced by $3.7 million, $4.2 million, $11.5 million, and $6.6 million and the authors found that, in their model, reduced medical costs steadily increased in 2015–2018 [37].

Akase et al. studied the hospitalization period and direct medical cost in patients using warfarin or NOACs after a cerebral embolism in Japan. They focused on costs from the first NOAC/warfarin treatment date to the discharge date from the hospital (for example, the fees for hospitalization, examinations, drugs, imaging). The total cost for patients in the NOACs’ group ($7151 ± 6228) tended to be lower than those in the warfarin group ($8950 ± 5891) (*p* = 0.054): due to the high cost of NOACs, the mean cost of a prescription for NOAC-treated patients was significantly higher than for warfarin treatment, the mean cost of laboratory tests for NOAC-treated patients was significantly lower, and the mean cost of hospitalization for NOAC-treated patients was lower than for warfarin-treated patients probably due to shorter hospital stays for NOAC-treated patients [38].

Mueller et al. conducted a retrospective analysis of an anonymized claims’ data set from three German Health insurance funds. They studied the effectiveness and safety of oral anticoagulation strategies (VKA or NOACs) in atrial fibrillation. Unlike previous studies, in this study, VKA therapy showed superiority over NOAC therapy for all outcomes (effectiveness and safety) in the real world. The authors compared these results with randomized clinical trials (RCTs) and showed that VKA therapy was more effective than NOAC therapy, whereas the analysis of safety outcomes led to inconclusive results. These results could be influenced by several factors such as different clinical characteristics of patients and common non-adherence to NOAC therapy. NOAC therapy was associated with higher event rates for the outcomes analyzed. In Germany, 94% of AF patients receive Phenprocoumon and not warfarin [39].

Degli Esposti et al. studied the health care resources used in patients treated with oral anticoagulants in Italy. They analyzed administrative databases that included about 2 million people from two Local Health agencies in north Italy. 

Health care resource use was studied in terms of drugs, hospitalizations, and outpatient specialist services and the average cost was €5156.13 for a VKA patient and €4630.57 for a NOAC patient for 12 months of follow-up [40].

These studies, apart from that by Mueller et al., showed that when NOACs are used instead of VKAs annual medical costs are reduced. 

### Local Drug Use Data

The Medicines Utilisation Monitoring Centre (OSMED) 2018 Report, produced by the Italian Drug Agency (AIFA), shows that Anatomical Therapeutic Chemical (ATC) Blood (B) drugs represent the fifth most-spent therapeutic category in Italy in 2018 (2082 million euros, 34.43€ per capita). The overall positioning of this category is mainly justified by the expenditure resulting from the purchase by public health facilities (26.52 euros per capita) (Table 1) [41].

Table 2 reports the total expenditure of the antiplatelet agents and anticoagulants’ subgroup for the year 2018. NOACs are the category with the largest increase compared to 2017 (up 27.6%), followed by ticagrelor (14.7%) and clopidogrel alone or in association (9.7%).

Indeed, apixaban, rivaroxaban, dabigatran, and edoxaban are the molecules with the greatest variation compared to 2017.

Also, the OSMED 2018 Report shows that 8.4% of patients treated with NOACs in Italy are residents in Puglia [41]. In November 2019, for the prevention of cerebral stroke and systemic embolism for patients with NVAF and the treatment and prevention of recurrences of DVT and PE in adults, respectively, 96,180 and 9708 patients were treated, while interrupted treatments were 8510 and 555, respectively [42]. In Puglia, health care is guaranteed by the presence of the Local Health Agency (LHA) and Hospital Company. Among them, LHA BT operates in the territory coinciding with the province of BT (Barletta-Andria-Trani) with a population of 391,224 inhabitants. The Agency guarantees hospital care through three direct-managed presidencies, divided into five hospital plexuses (hospitals of Andria, Canosa, Barletta, Bisceglie, and Trani), with the availability of 548 beds for ordinary stays, eight for “day-surgery”, and 35 for “day-hospital” [43].

**Figure 1:**
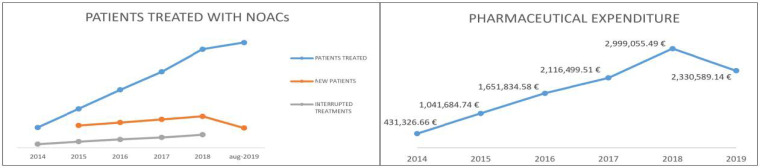
Patients treated in LHA BT and pharmaceutical expenditure trend.

Table 3 shows the data, compiled through the Regional Health Information System–Edotto, relating to LHA BT in the period January 2014–August 2019. In particular, the number of new patients treated annually and the number of patients who discontinued treatment are shown [44]. The number of interrupted treatments is high due to patients who started NOACs treatment for the prevention of venous thromboembolic events (after elective hip or knee replacement surgery) and patients taking NOACs for a short-term treatment (at least three months) based on transient risk factors of DVT and PE, under the available guidelines. Figure 1 shows the trend of pharmaceutical spending supported by LHA for the purchase of NOAC (dispensed through the “distribuzione per conto”—DPC channel) in the period January 2014–August 2019 [45].

Effectiveness and safety data extrapolated from clinical trials are increasingly being confirmed by encouraging data in the Real World. It is, therefore, necessary to evaluate the potential translation of Clinical trial data to the local data to improve the cost/effectiveness of these drugs.

So, because of the introduction of NOACs on the Italian pharmaceutical market and the extensive use in clinical practice, the purpose of this work was to assess the economic and health impact in the LHA BT, a local health authority in southern Italy.

## 2. Materials and Methods

In this retrospective study, a group of naïve patients in treatment with a NOAC and a group of VKA-treated patients as a control group were considered. We extrapolated anonymized data from the Regional Information System Edotto. Patients in treatment for the first time with a NOAC in the year 2017 were compared with patients taking a VKA (warfarin, acenocoumarol).

For these patients the study assessed:

- therapeutic continuity in the year 2018;
- pharmaceutical spending (total and per capita) for the years 2017 and 2018;
- any hospitalizations, discharge diagnosis, and related costs;
- access to the emergency room (ER); and
- deaths.

In the work, principal or secondary discharge diagnoses, classified according to the International Classification of Diseases (ICD9) and related to treated pathologies or relative clinical events (Table S1 in Supplementary Material), were considered. The data were stratified into seven categories: *myocardial infarction, brain hemorrhage, brain thrombosis and embolism, ischemic events, embolism and vascular thrombosis, GI hemorrage and pulmonary embolism*.

Drug costs were evaluated using the Italian National Health System (NHS) purchase price. Hospitalization costs were determined using the DRG (diagnosis-related group) tariffs reimbursed by NHS.

Data were analyzed with intra- and inter-group analysis.

Descriptive statistical analyses were made by “RStudio” software v. 3.6.2. Relative Risk (RR) and Odds Ratio (OR) were expressed as value and 95% Confidence Interval (CI).

## 3. Results

### 3.1. Patients in Treatment with NOACs

**Figure 2:**
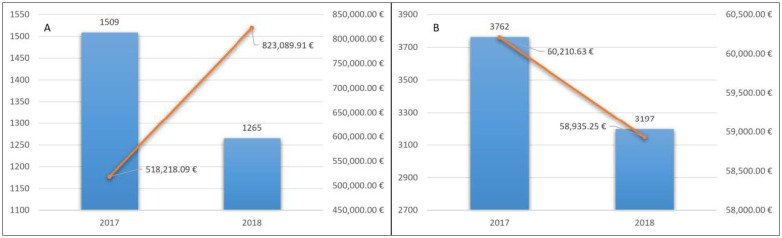
Naïve patients treated with NOACs (**A**) and with VKA (**B**) and related pharmaceutical spending.

In 2017 LHA BT counted 1509 patients treated for the first time with NOACs. The total cost for the delivery of drugs was 518,218.09 euros, with an average cost of 343.42 euros (Figure 2A). 

**Figure 3:**
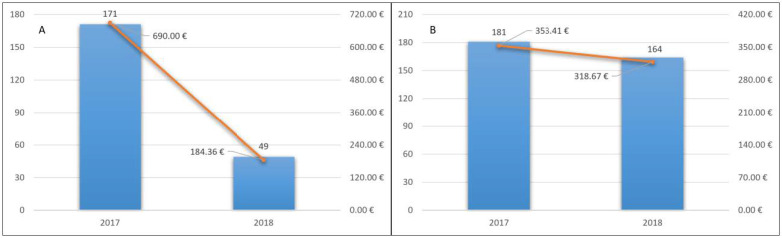
Hospitalization cost for a patient treated with NOACs (**A**) and VKA (**B**) and hospitalizations’ trend.

Of these patients, 171 (11.3%) were hospitalized in 2017 and the total number of hospitalizations was 235. The total cost of hospitalizations was 1,041,216.54 euros. The average cost for inpatient was 6088.99 euros. The average cost for a patient treated with NOACs was 690 euros (Figure 3A).

Patients with access to the emergency room were 109 with 73 deaths.

In 2018, 1265 (84%) patients continued therapy. This confirmed the good long-term tolerability of these drugs. Pharmaceutical expenditure for them was 823,089.91 euros, with an average cost for treated patients of 650.66 euros (Figure 2A).

In 2018, 49 (3.9%) of these patients were hospitalized, with a total of 64 hospitalizations. The total cost of hospitalizations was 278,198.08. The average cost for inpatient was 5677.51 euros. The average cost for a patient treated with NOAC was 184.36 euros (Figure 3A). Twenty-five accesses to the emergency room and 76 deaths occurred.

### 3.2. Patients in Treatment with VKA

In 2017, LHA BT counted 4420 patients with at least a dispensation of a VKA (warfarin, acenocoumarol). Patients included in the analysis were 3762 because 658 switched to an NOAC treatment and were excluded.

The total cost for the delivery of drugs was 60,210.63 euros, with an average cost for the treated patients of 1600 euros (Figure 2B).

Of these patients, 181 (4.8%) were hospitalized in 2017 and the number of hospitalizations was 246. The total cost of hospitalizations was 1,329,526.70 euros. The average cost for inpatient was 7345.45 euros. The average cost for a treated patient with VKA was 353.41 euros (Figure 3B).

Patients with access to the emergency room were 90 and 232 deaths.

In 2018, 3197 (85%) patients continued therapy. Pharmaceutical expenditure was 58,935.25 euros, with an average cost for the treated patients of 18.43 euros (Figure 2B).

Similarly, 164 (5.13%) patients were hospitalized, with a total of 205 hospitalizations. The total cost of hospitalizations was 1,018,782.97. The average cost for inpatient was 6212.09 euros. The average cost for a patient treated with VKA was 318.67 euros (Figure 3B). Sixty-eight accesses to the emergency room and 259 deaths occurred.

### 3.3. Analysis of Discharge Diagnoses 

Hospitalization data were stratified by analyzing as an outcome the diagnoses: myocardial infarction, cerebral hemorrhage, thrombosis, cerebral embolism, ischemic events, embolism, vascular thrombosis, GI hemorrhage, and pulmonary embolism. The incidence, expressed as the number of events/100 patients per year, was calculated for each of them and reported in Tables S2–S5 in SI.

We observed an evident reduction, in patients in long-term treatment with NOACs, of the incidence (number of events/100 persons Year) of major discharge diagnoses (Tables S2 and S3 in Supplementary Material). For patients treated with VKAs, the incidence remained constant (Tables S4 and S5 in Supplementary Material) except for brain hemorrhage that increased from 0.32 in 2017 to 0.75 in 2018. Moreover, the relative risk (RR) with the 95% confidence interval (CI), for each group and each discharge diagnosis, was calculated (Table 4 and Table 5). After only one year, the long-term treatment with NOACs resulted in a greater reduction of the RR for the discharge diagnoses analyzed, compared to VKAs.

Table 6 shows the Odds Ratio (OR) of the main discharge diagnoses analyzed, calculated by considering the number of cases that occurred in 2018 in both treatment groups (Table 4 and Table 5).

Except for myocardial infarction and ischemic events, where the OR was 1, for all other diagnoses considered, the OR values were less than 1 and, in particular, for vascular and thrombotic events (arterial and venous) the OR was 0.16.

These data are in line with the RCTs’ results and the Real-World Data reported in SI.

The number of deaths in both cohorts remained almost constant over the two years of analysis.

## 4. Discussion

### 4.1. Present Findings

These data are aimed at allowing the best management of patients with NVAF and VTE in a phase of health emergency that is still affected by the critical issues related to the logistical and organizational difficulties of access to the company’s specialized outpatient clinics. Anyway, they do not modify some critical issues regarding the choice of the appropriate drug depending on whether the patient is at thromboembolic risk or risk of bleeding. This is one of the reasons why old anticoagulants, which still cover about 25% of the market, are still used.

Particular attention should be paid to certain patient populations, including elderly patients, patients with kidney damage, and patients with cancer in the active phase, as a wrong treatment choice would cause some complications for these patients and would significantly burden the already limited resources available.

Due to their favorable safety profile, fixed dosages, and lower monitoring of International Normalized Ratio (INR), NOACs are considered the most attractive choice for elderly patients.

In the authoritative clinical trials, patients aged 80 and over were represented in a small percentage, which undermined the translation of the results to the elderly population. The only trial where a large number of elderly patients (about 40%) were enrolled is the “Effective Anticoagulation with Factor Xa Next Generation in Atrial Fibrillation—Thrombolysis in Myocardial Infarction 48” (ENGAGE-AF TIMI 48). Some substudies of “Apixaban for Reduction of Stroke and Other Thromboembolic Events in Atrial Fibrillation” (ARISTOTELE) and ENGAGE-AF TIMI 48 studies showed that the clinical benefit of apixaban and edoxaban in reducing the risk of major bleeding is also preserved in elderly patients.

On the contrary, further analyses on the authoritative studies of dabigatran showed that both doses of dabigatran increased the risk of bleeding in patients over 75 years of age where there is a decline in kidney function that could lead to an increase in the drug’s plasma concentrations [27,45].

In a retrospective study of a cohort of 11,760 patients aged 75 years with atrial fibrillation, Alnsasra and colleagues showed that the greatest clinical benefit in elderly patients was obtained through the administration of warfarin in the presence of a time in therapeutic range (TTR) ≥ 60% and, therefore, with good control of therapy and with high-dose NOACs as expected by summary of product characteristics (SmPC) [45].

Patients with severe kidney damage and those requiring transplantation were excluded from the trials. To date, few real-world studies have assessed the efficacy and safety of NOACs based on kidney function, but all showed that these are at least as effective as warfarin in terms of safety [31,32,46,47,48].

Cancer patients have a 4 to 7 times higher risk of venous thromboembolism (VTE), specifically of deep vein thrombosis (DVT) and pulmonary embolism (PE). NOACs have been approved for the treatment of VTE in the population. Meta-analysis networks suggest that they could have similar effectiveness and safety even compared to LMWH in the treatment of cancer-associated thrombosis [49,50,51].

NOACs and LMWH have recently been compared in RCTs. These trials compared edoxaban and rivaroxaban with dalteparin in patients with active cancer, most of which were metastatic. In patients treated with NOACs, a lower incidence of VTE recurrences was found, but a higher incidence of major bleeding and non-clinically significant bleeding compared to LMWH was found [36,52].

In the pandemic COVID-19 population, low-molecular-weight heparin is, however, recommended for critically ill patients in China [53]. Some groups are initiating treatment-dose LMWH in patients with D (fibrin D fragment)-dimer levels >3000 μg/L. Clinical trials evaluating the impact of anticoagulation with different dosages of LMWH and/or unfractionated heparin on survival are in progress (NCT04345848; NCT04344756) [54].

An elevated risk of thrombosis was observed in the pediatric population in hospital, associated with central venous access devices (CVAD). VTE occurs in this population affected by coronary heart disease (CHD), surgery, infection, malignancy, prematurity, use of oral contraceptives, immobilization, or in the inherited thrombophilia and the presence of antiphospholipid antibodies.

Unfortunately, VTE treatment in pediatric patients is not evidence-based and the treatments are based on adult studies.

It should be of note that the standard of care for the VTE treatment in children includes LMWH, unfractionated heparin, and VKA, depending on the hospital and the existence of systemic thrombolysis and/or physical thrombectomy that may be performed by expert people [55].

Several controlled trials providing pharmacokinetics/pharmacodynamics (PK/PD), efficacy, and safety on these drugs in children are underway and some were completed. Preliminary data from these studies indicate that NOACs have consistent PK/PD relationships and may show at least comparable efficacy and safety as LMWH and VKA overall in pediatric age groups. These studies will help to establish evidence-based guidelines for the treatment and prevention of thromboembolic events in children and adolescents with various underlying conditions providing specific pediatric formulations [56,57].

### 4.2. Implications for Health Policy Makers

Our data show the significant economic impact of NOACs on the Regional Health System, due to the high cost of these therapies compared to traditional VKAs.

We found that in 2017 in LHA BT, naïve patients treated with NOACs were 1509. The average cost of drug therapy for the treated patient was 343.42 euros. In 2018, 1265 continued the treatment and the average cost for the treated patients was 650.66 euros.

In 2017, 3762 patients were treated with AVK and included in the analysis. The average cost of drug therapy for the treated patients was 16.00 euros. In 2018, 3197 continued treatment, and the average cost for the treated patients was 18.43 euros.

**Figure 4:**
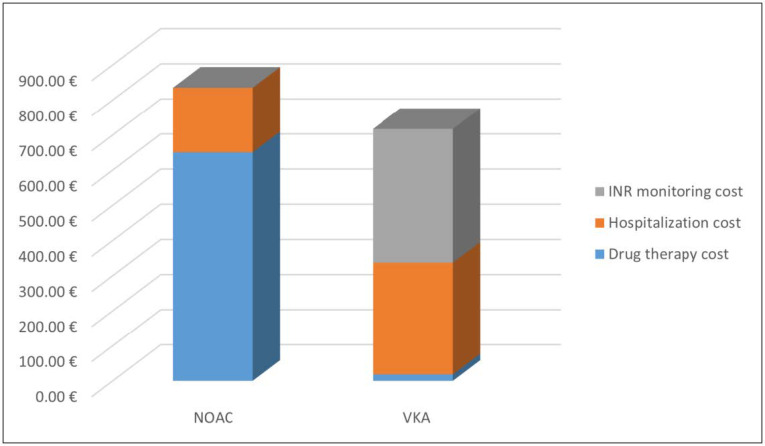
Average cost for patients treated with oral anticoagulants in 2018.

The average cost of hospitalizations decreased from 690 euros in 2017 to 184.36 euros in 2018, for patients treated with NOACs. The average cost of hospitalizations for the patients treated with VKAs was 353.41 euros in 2017 and 318.67 euros in 2018.Considering the data of 2018, for patients adhering to long-term therapy, the total direct health cost for the patients treated with NOACs was 835.02 euros, while for the patients treated with VKAs was 337.10 euros. This difference is fully compensated because VKAs treatment requires continuous INR monitoring and the cost is around 380 euros per year, as reported by Pradelli et al. [58]. Thus, the total cost for the treated patients with VKAs was 717.10 euros (Figure 4).

On the other hand, treatment with NOAs showed a marked reduction in the number of hospitalizations and this, taking into account the average cost of a hospital stay (4346.85 euros for NOAs and 4969.67 euros for VKAs) and the entire population of patients treated with NOAs, could generate, in the long term, saving for the Regional Health System.

Our findings are in line with the results of the study by Amin et al., in which, based on the results of clinical trials, medical costs were consistently reduced by the use of NOACs instead of VKAs [37].

Our data are also in line with the study by Akase et al., in which the authors found a higher cost for NOAC prescriptions but lower costs due to hospitalization mainly due to a shorter hospital stay [38]. Finally, our data almost overlap with the findings of Degli Esposti et al., who analyzed medical costs in two LHAs in northern Italy [40].

Due to the cost of therapy, a difference in health care costs was found, but this will tend to decrease in the coming years. Indeed, in 2019, AIFA Prices and Refunds Committee renegotiated the cost of treatment with NOAs, thus ensuring the best therapeutic outcomes that patients receive from this class of drugs with an estimated resource savings of approximately 60 million euros [59]. Finally, it is necessary to consider the recent publication of AIFA Note 97, which, initially due to the COVID-19 emergency, allowed the renewal of treatment plans for patients treated with NOAC. Moreover, Note 97, now definitively adopted, allows the prescription by both general practitioners and specialists for the treatment of non-valvular atrial fibrillation (FANV). With the new note, vitamin K antagonists (warfarin, acenocoumarol) will also follow the same prescriptive rules [60].

### 4.3. Clinical Implications

Clinically, the situation completely reverses. In fact, for the patients treated with VKAs the number of hospitalizations was almost constant, 246 in 2017 and 205 in 2018. For patients treated with NOACs, however, the number of hospitalizations decreased from 235 in 2017 to 64 in 2018. The incidence, RRs, and ORs calculated for the discharge diagnosis analyzed, according to the Real-World Data, confirm the safety and effectiveness of this class of drugs and highlight the reason for the wide use in clinical practice.

### 4.4. Limitation of Our Data

The aim of the study was the evaluation of the impact of the introduction of NOACs into clinical practice in LHA BT. For this reason, patients already being treated with NOACs were excluded from the analysis and it was possible to foresee that they had already achieved a status quo in terms of health costs and clinical events. Thus, variations were difficult to observe. The aggregate data of patients treated regardless of the therapeutic indication were considered and this represents a limitation of the analysis.

## 5. Conclusions

The study shows that the treatment of naïve patients treated with NOACs, compared to traditional VKAs therapy, involves, for LHA BT, an increase in expenditure of about 100 euros for a patient, considering only direct health costs. However, treatment with NOACs reduced the number of hospitalizations in our regional area with improved safety and effectiveness.

Of course, NOACs will not be able to completely replace VKAs in clinical practice, as they have therapeutic indications excluded from NOACs, such as the prevention of stroke and systemic embolism in patients with atrial fibrillation and mechanical heart valves.

In conclusion, the challenge of health systems is the correct management of the resources available, while that of clinicians is the correct use in the Real World, the correct selection of patients and dosages, and the monitoring of therapeutic adherence. Attention to special populations is, however, needed to improve the safety and efficacy of NOACs.

## Figures and Tables

**Table 1:** National Health System (NHS) cost per capita for first ATC Levels: 2018–2017 comparison (data from the OSMED 2018 Report). A: gastrointestinal system and metabolism; B: blood and hematopoietic organs; C: cardiovascular system; D: dermatological; G: genito-urinary system and hormones; H: systemic hormonal preparations, excluding sex hormones; J: systemic-use antimicrobial; L: antineoplastic drugs and immunomodulatory; M: musculoskeletal system; N: central nervous system; P: pesticides; R: respiratory system; S: sense organs; V: various.

I ATC Level	Per Capita Expenditure Conventional Assistance (a)	Δ%18–17	Per Capita Expenditure Public Health Company (b)	Δ%18–17	a + b	Δ%18–17
L	4.1	4.3	89.46	9.7	93.56	9.5
C	48.98	−8.7	4.59	−7	53.57	−8.6
J	13.07	−0.6	35.16	−21.2	48.23	−16.6
A	33.01	0.6	13.87	10	46.88	3.2
B	7.91	−2.2	26.52	0.5	34.43	−0.1
N	22.53	0.4	6.94	1.1	29.47	0.6
R	16.23	−0.8	3.05	44.9	19.28	4.4
M	5.71	−6	3.11	103.7	8.82	16
H	3.98	4.9	4.62	0.3	8.6	2.4
G	5.64	−14.6	1.57	−15.4	7.21	−14.8
S	3.81	−0.1	2.81	−7.9	6.62	−3.6
V	0.15	4.6	5.33	3.7	5.48	3.7
D	1.13	19.1	0.39	10	1.52	16.6
P	0.22	3.1	0.03	−5.3	0.25	2.2
Total	166.46	−3.2	197.45	0.9	363.91	−1

**Table 2:** Total pharmaceutical expenditure of the antiplatelet agents and anticoagulants’ subgroup for the year 2018 (data from the OSMED 2018 Report).

Antiaggregants and Anticoagulants	Total Expenditure (in Million euros)	% on NHS Expenditure	Per Capita Expenditure	Δ%18–17	DDD/1000 Inhab per day	Δ%18–17
New Oral Anticoagulants	445.2	2.0	7.36	17.8	9.4	27.6
Low molecular weight heparins	256.3	1.2	4.24	−13.1	8.9	−3.8
Antiplatelet drugs excluding clopidogrel, prasugrel and ticagrelor	133.6	0.6	2.21	2.3	58.9	−0.7
Clopidrogrel individually or in association	69.8	0.3	1.15	4.5	10.2	9.7
Antiaggregants and vasodilator effects	55.6	0.3	0.92	2.8	<0.05	−0.9
Ticagrelor	52.1	0.2	0.86	15.2	1.0	14.7
Fondaparinus	16.4	0.1	0.27	−3.2	0.5	−2.0
Vit K antagonists	11.4	0.1	0.19	−10.1	4.6	−10.1

**Table 3:** Summary of patients treated in LHA BT in the period January 2014–August 2019.

Year	Treated Patients	New Patients for Year	Discontinued Patients
**2014**	1079		203
**2015**	2063	1186	337
**2016**	3066	1340	448
**2017**	4017	1509	557
**2018**	5208	1665	700
**August 2019**	5562	1054	

**Table 4:** RR for major discharge diagnoses analyzed: NOACs.

ICD Diagnosis of NOAC Discharge	Cases 2017	Cases 2018	Tot Cases	RR	CI 95% (RR)−	CI 95% (RR)+
Myocardial infarction	63	15	78	0.28	0.25	0.41
Brain hemorrhage	4	6	10	1.79	0.13	1.79
Brain thrombosis and embolism	52	10	62	0.23	0.21	0.26
Ischemic events	14	4	18	0.34	0.26	0.43
Embolism and vascular thrombosis	24	1	25	0.05	0.046	0.06
GI Hemorrhage	11	7	18	0.76	0.53	1.11
Pulmonary embolism	29	2	31	0.08	0.07	0.09

**Table 5:** RR for the main discharge diagnoses analyzed: VKAs.

Icd Diagnosis of AVK Discharge	Cases 2017	Cases 2018	Tot Cases	RR	CI 95% (RR)−	CI 95% (RR)+
Myocardial infarction	65	42	107	0.76	0.66	0.89
Brain hemorrhage	12	24	36	2.35	1.48	3.71
Brain thrombosis and embolism	30	30	60	1.18	0.92	1.52
Ischemic events	17	10	27	0.69	0.52	0.92
Embolism and vascular thrombosis	23	16	39	0.82	0.63	1.06
GI Hemorrhage	36	28	64	0.92	0.74	1.15
Pulmonary embolism	11	8	19	0.86	0.59	1.26

**Table 6:** Odds ratio (NOACs vs. VKAs) for major discharge diagnoses analyzed.

ICD Diagnosis of Discharge 2018	Incidence/100 pers Year NOACs	Incidence/100 pers Year VKAs	OR NOACs/VKAs	CI 95% OR−	CI 95% OR+
Myocardial infarction	1.19	1.31	1	0.55	1.8
Brain hemorrhage	0.47	0.75	0.63	0.26	1.55
Brain thrombosis and cerebral embolism	0.79	0.94	0.88	0.43	1.8
Ischemic events	0.32	0.31	1	0.31	3.19
Embolism and vascular thrombosis	0.08	0.5	0.16	0.02	1.2
GI Hemorrhage	0.55	0.88	0.66	0.29	1.51
Pulmonary embolism	0.16	0.25	0.66	0.14	3.1

## Data Availability

Data are not publicly available.

## Supplementary Materials

The following are available online at https://www.mdpi.com/1999-4923/13/2/252/s1, Supplementary material 1. Pharmacological properties, clinical trials, and real-world data. Table S1: ICD9-CM codes considered in the analysis. Table S2: Aggregated diagnosis of resignation 2017 and related incidence: patients treated with NOACs. Table S3: Aggregated diagnosis of resignation 2018 and related incidence: patients treated with NOACs. Table S4: Aggregated diagnosis of resignation 2017 and related incidence: patients treated with VKAs. Table S5: Aggregated diagnosis of resignation 2018 and related incidence: patients treated with VKAs.
