# Supplementary material for: Analysis of the Introduction in Clinical Practice of New Oral Anticoagulants in Local Health Agency BT: Translation of the Clinical Trial Data to a Local Health Care Area
Source: Pharmaceutics. 2021 Feb 11;13(2):252. doi: 10.3390/pharmaceutics13020252 (PMC7918324; doi:10.3390/pharmaceutics13020252)
Supplement: Supplementary file 1 [file pharmaceutics-13-00252-s001.pdf]

pharmaceutics

MDPI

Supplementary Material: Analysis of the Introduction in Clinical Practice of New Oral Anticoagulants in Local Health Agency BT: Translation of the Clinical Trial Data to a Local Health Care Area

Gianluca Grimaldi, Domenica Ancona, Domenico Tricarico, Paolo Stella, Cataldo Procacci, Antonio Germinario, Vito Bavaro, Vito Montanaro and Alessandro Delle Donne

Citation: Grimaldi, G.; Ancona, D.; Tricarico, D.; Stella, P.; Procacci, C.; Germinario, A.; Bavaro, V.; Montanaro, V.; Delle Donne, A. Analysis of the Introduction in Clinical Practice of New Oral Anticoagulants in Local Health Agency BT: Translation of the Clinical Trial Data to a Local Health Care Area. *Pharmaceutics* **2021**, *13*, 252. https://doi.org/10.3390/pharmaceutics13020252

Academic Editor(s): Lucio Barile
Received: 16 December 2020
Accepted: 8 February 2021
Published: 11 February 2021

Publisher's Note: MDPI stays neutral with regard to jurisdictional claims in published maps and institutional affiliations.

Copyright: © 2021 by the author. Licensee MDPI, Basel, Switzerland. This article is an open access article distributed under the terms and conditions of the Creative Commons Attribution (CC BY) license (http://creativecommons.org/licenses/by/4.0/).

Supplementary Material 1. Pharmacological properties, Clinical Trials, and Real-World Data

1.1. Dabigatran

Dabigatran etexilate is an oral anticoagulant with a non-peptide structure of synthetic origin that inhibits Factor IIa (thrombin) preventing the formation of fibrin. Due to its high hydrophilic properties, it is administered as a prodrug rapidly metabolized to dabigatran by hepatic and intestinal carboxyl and plasma esterases [1].

The absolute bioavailability of dabigatran etexilate after oral administration is about 6.5% with an important variability in absorbed quota [2]. Absorption increases in an acidic environment so are formulated in the presence of tartaric acid. Co-administration with antiH2 drugs and proton pump inhibitors consequently leads to a decrease in absorption of 12 and 30% respectively. Taking with a meal rich in fat may delay the time needed to reach Cmax, however, has no significant effect on bioavailability, AUC, or plasma concentration [3]. Dabigatran has a rapid action, a peak plasma concentration after about 2 hours after oral administration, and a half-life of about 12-17 hours. Dabigatran is not metabolized in the liver [4,5].

Dabigatran is a substrate of the glycoprotein P (P-gp), a protein present in the intestine that influences its absorption. Interactions with powerful inducers (such as rifampicin, carbamazepine, St. John's wort) or inhibitors (such as fluconazole, ketoconazole, amiodarone, verapamil, clarithromycin, quinidine) of P-gp can modify the thrombotic and hemorrhagic risk of patients in treatment [4,5].

It shows a poor binding to plasma proteins (34–35%) and a renal clearance that makes it the only one that can be hemodialysis. Renal excretion accounts for 80% of its total clearance, the remainder is conjugated with glucuronic acid and excreted by bile. It is contraindicated in patients with creatinine clearance (ClCr) < 30 mL/min and should be administered at reduced doses in those with ClCr between 30 and 50 mL/min, in patients with severe renal failure there is a 6.3-fold increase in AUC and a half-life of 28 hours compared to healthy subjects. In all patients and especially in the elderly (>75 years), since renal impairment may be frequent in this age group, renal function must be assessed by calculating creatinine clearance [2,4]. Dabigatran is now marketed as capsules in dosages of 75mg 110mg and 150mg.

Dabigatran authorized indications are:

- primary prevention of venous thromboembolism episodes in orthopedic surgery (initial dose of 110 mg on the day of surgery, a maintenance dose of 220 mg for 10-35 days).
- Stroke and systemic embolism prevention in adult patients with nonvalvular atrial fibrillation (NVAF), with one or more of the following factors risk (300 mg per day)
- Treatment of deep vein thrombosis (DVT) and pulmonary embolism (PE) and prevention of recidivism of DVT and PE in adults (300 mg per day after 5 days of treatment with injecting anticoagulant).

The RE-LY study compared two doses of Dabigatran (150 and 110 mg) to warfarin, both doses showed a significant reduction in the risk of hemorrhagic stroke. However, an increased risk of myocardial infarction (MI) was observed with both dosages (110 mg RR 1.35, 150 mg RR 1.38) [6].

A retrospective sub-study of the trial was conducted to examine MI and myocardial ischemic events. It showed that the increased risk of MI was unremarked. However, further meta-analyses showed mixed results, thus emphasizing the need for greater post-marketing surveillance.[7] The meta-analysis of Natios et al. of 5 Real World studies showed the absence of significant differences between dabigatran and warfarin (Hazard Ratio HR 0.96) in MI risk. This was further supported by a study investigating the risk of MI with the use of NOACs and VKAs. This study was based on indirect comparisons between NOAC and no significant differences in MI risk were found, contrary, relevant reductions in the risk of MI with all NOACs were observed compared to warfarin [8].

Natios and colleagues showed that the risk of GI bleeding is significantly higher with dabigatran than with warfarin (HR 1.20), but an overall reduction in mortality with dabigatran (HR 0.63) mainly due to reduced intracranial bleeding was observed [9].

1.2. Rivaroxaban

Rivaroxaban is a direct oral inhibitor of factor Xa, competitively inhibiting both free and clot-related factor Xa. Rivaroxaban is rapidly absorbed after oral administration and Cmax is reached after 2–4 hours. Oral absorption is almost complete and bioavailability is high (80–100%), regardless of fasting or food intake. The pharmacokinetics of rivaroxaban is linear up to about 15 mg once a day, dosages ≥15 mg have a reduced absorption on fasting, which is restored after food-associated intake [10].

The binding to plasma proteins is high (92–95 %, mainly albumin). The distribution volume is approximately 50 L. Due to the high plasma protein binding, rivaroxaban is not dialysable. About 2/3 of the administered dose of rivaroxaban undergo metabolic degradation with subsequent renal and fecal elimination. The remaining 1/3 is excreted directly via the kidney. Rivaroxaban is metabolized by CYP3A4, CYP2J2, and by CYP independent mechanisms. It is a substrate of P-gp and Bcrp (breast cancer resistance protein). Substances with combined inhibitory actions such as many antifungals drugs and protease inhibitors significantly increase drug concentrations and hemorrhagic risk [10,11].

Rivaroxaban is contraindicated in patients with liver disease associated with coagulopathy and clinically relevant hemorrhagic risk, including cirrhotic patients. Increased exposure to rivaroxaban has been found in patients with reduced renal function. Rivaroxaban pharmacokinetics show low intra- and inter-individual variability, making routine monitoring unnecessary [12]. Rivaroxaban is now marketed as coated tablets in dosages of 2.5mg, 10mg, 15mg, and 20 mg.

Rivaroxaban authorized indications are:

- Prevention of atherothrombotic events in adult patients after an acute coronary syndrome (ACS) with elevated cardiac biomarkers (2.5 mg twice a day with acetylsalicylic acid-ASA alone or with ASA and clopidogrel or ticlopidine).
- Prevention of events atherothrombotic in adult patients, at high risk of ischemic events, presenting coronary artery disease (cCAD) or symptomatic peripheral artery disease (PAD) (2.5 mg twice a day with ASA).
- Prevention of venous thromboembolism (VTE) in adult patients undergoing replacement surgery elective hip or knee (10 mg per day for 2-5 weeks).
- Deep vein thrombosis (DVT) and pulmonary embolism (PE) treatment and prevention of recurrences of DVT and EP in adults (15 mg twice a day for the first three weeks, followed by a dose of 20 mg once daily for the continuation of the treatment and the prevention of recidivism of DVT and PE).
- Prevention of Stroke and systemic embolism in adult patients with NVAF with one or more risk factors, such as congestive heart failure, hypertension, age ≥75 years, diabetes mellitus, previous stroke, or transient ischemic attack (20 mg per day).

The results of the Real World studies for rivaroxaban are in agreement with those of the authorizing Rocket-AF study [13]. When compared with other NOACs, rivaroxaban shows a higher risk for major bleeding, GI bleeding, and mortality and a comparable risk of intracranial bleeding compared to apixaban [18,19], a higher risk of major bleeding, GI bleeding, mortality, and intracranial bleeding compared with dabigatran, mainly in the elderly population [8,14].

1.3. Apixaban

Apixaban is a direct, reversible, and highly selective inhibitor of Factor Xa. Pharmacokinetic studies have shown that the oral bioavailability of apixaban is 50%. It is rapidly absorbed in the distal tract of the small intestine and ascending colon (Cmax occurs 3–4 hours after administration). Oral bioavailability is not affected by the simultaneous intake of food, fat-rich meals, and antiacid drugs. The half-life is 8–15 h, plasma protein binding is about 87% and the distribution volume is 21 L. Apixaban shows an intra- and inter-individual variability of 20–30% [15,16].

Apixaban has several elimination pathways, it is metabolized in the liver by the CYP3A4 and CYP3A5. About 25% of the administered dose has been detected as metabolites, the remainder is found in the feces; renal excretion accounts for 27% of total clearance and occurs through active transport mediated by P-gp and BCRP. The excretion is renal, biliary, and direct intestinal excretion [15,17].

Given the biotransformation and elimination pathways, the simultaneous intake of apixaban with potent dual cytochrome CYP3A4 and P-gp inhibitors is not recommended. Similarly, the simultaneous intake of cytochrome CYP3A4 and P-gp inducers is not recommended. These drugs may significantly increase or reduce exposure to the drug causing imbalances in the thrombotic/hemorrhagic risk ratio [18].

No impact of impaired renal function on the plasma peak of apixaban has been observed, there has been an increase in drug exposure correlated with a decrease in CLCr. In individuals with mild, moderate, and severe renal impairment, AUC increased by 16, 29, and 44% respectively. In individuals with end-stage renal disease, after administration of 5mg, apixaban's AUC increased by 36%; hemodialysis initiated two hours after administration decreased AUC by 14%, making it unlikely that hemodialysis is an effective system for managing overdose [15,18]. Apixaban is now marketed as coated tablets in dosages of 2.5 mg and 5 mg.

Apixaban authorized indications are:

- Prevention of venous thromboembolic events (VTE) in adult patients undergoing elective hip or knee replacement surgery (2.5 mg twice a day for 10–38 days)
- Prevention of stroke and systemic embolism in adult patients with non-valvular atrial fibrillation (NVAF), with one or more risk factors, such as a previous stroke or ischemic attack transient (TIA), age ≥75 years, hypertension, diabetes mellitus, symptomatic heart failure (NYHA Class ≥ II) (5 mg twice a day for 10–38 days)
- Treatment of deep vein thrombosis (DVT) and pulmonary embolism (PE) and prevention of recurrences of DVT and EP in adults (10 mg twice a day for 7 days followed by 5 mg twice a day for the treatment, 2.5 mg twice a day for prevention after 6 months of anticoagulant treatment for DVT or PE).

2 large trials were performed for the use of apixaban in patients with NVAF: AVERROS study and ARISTOTELE study.

The AVERROS study was prematurely discontinued after about 1 year due to the clear superiority of apixaban over aspirin in reducing the risk of stroke, while no significant statistical differences regarding the risk of major and GI bleeding, intracranial hemorrhage, and mortality were observed [19].

In the ARISTOTELE study, apixaban was found to be higher than warfarin in reducing the risk of hemorrhagic stroke (HR 0.51), major bleeding (HR 0.69), and death (HR 0.89) [20].

The ARISTOPHANES study, a large Observational study conducted on 5 U.S. databases, showed that apixaban, compared with rivaroxaban, is superior for all analyzed outcomes (stroke, hemorrhagic stroke, systemic embolism, major bleeding, GI bleeding, intracranial hemorrhage, and other types of bleeding) except for ischemic stroke where no significant difference was detected between them. Compared to dabigatran, apixaban was more effective in reducing the risk of stroke, ischemic stroke, major bleeding, and GI bleeding and not lower for hemorrhagic stroke, intracranial hemorrhage, and other types of bleeding [21].

Tepper et al. compared the hemorrhagic risk of patients with NVAF treated with NOACs. Researchers identified more than 60,000 adult patients who were treated with NOACs or who switched from warfarin to a NOAC. In this work were studied: 8785 patients with FA treated with apixaban, 20,963 with dabigatran, and 30.529 with rivaroxaban. The percentage of patients who discontinued warfarin to take a NOAC in each group were: 17.3% in the apixaban group, 15.7% in the rivaroxaban group, and 4.4% in the dabigatran group. In the group treated with apixaban, patients more frequently took antiplatelet drugs, more often had comorbidities, and had slightly higher CHA2DS2-VASc and HAS-BLED scores than patients treated with dabigatran or rivaroxaban. While most endpoints were similar, there was a significantly lower risk of GI bleeding with apixaban than dabigatran. Between apixaban and rivaroxaban, the adjusted risk of each type of hemorrhage was significantly higher with the latter (major bleeding HR 1,34, not-major but clinically significant hemorrhages HR 1.39) [22].

Deitelzweig et al. conducted an analysis using data from another U.S. health database, the PharMetrics Plus. The authors assessed the frequency of bleeding, hospitalization for all causes, and economic outcome in a sample of U.S. patients representative of the POPULATION with FA. Altogether, 9150 patients with NVAF who were naïve to oral anticoagulant therapy and had recently started treatment based on a NOAC were considered. 71.4% of patients were male and 84% of cases started therapy in 2013. The average age was 63.4 years and patients in the apixaban group were significantly older ($p = 0.0170$). The average value of the CHADS2 score was 1.8. Patients treated with dabigatran had a slightly lower HAS-BLED score and lower healthcare costs than patients receiving apixaban or rivaroxaban. When compared with apixaban, treatment with rivaroxaban and dabigatran was associated with an increased risk of hemorrhage (HR 1.8 $p < 0.0001$ and HR 1.27 respectively). Patients treated with rivaroxaban and dabigatran also showed a higher risk of hospitalization for all causes than apixaban (HR 1.60 and HR 14 respectively). Patients with apixaban generated lower overall costs ($ 3581) than those in dabigatran therapy ($ 4236; $p < 0.0001$) and rivaroxaban ($ 4144; $p < 0.0001$) [23].

1.4. Edoxaban

Edoxaban is a direct, highly selective, and reversible inhibitor of factor Xa. It presents a rapid onset of action within 1-2 h and effects are predictable and dose-related. Edoxaban after oral administration has an absolute bioavailability of about 62%, food increases Cmax but has a minimal effect on total concentration. It is poorly soluble at pH ≥ 6, co-administration with PPI had no significant effect on exposure to edoxaban.

The average distribution volume is 107 L, the binding to plasma proteins is about 55%. Edoxaban is metabolized by hydrolysis, conjugation, oxidation by CYP3A4 and CYP3A5; 3 active metabolites are known of which only the one obtained by hydrolysis is active.

Edoxaban is a substrate of P-gp, while its active metabolite is a substrate of the OATP1B1 capture transporter. Co-administration with P-gp inhibitors (such as cyclosporine, ketoconazole, quinidine, and verapamil) has resulted in increased plasma concentrations of the drug and some cases require administration at a reduced dose. Co-administration with P-gp inducers (such as rifampicin) has resulted in a reduction in the mean AUC and half-life of edoxaban and may reduce pharmacological effects.

Renal clearance accounts for approximately 35% of the total dose clearance, metabolism, and biliary clearance make up the remainder. In patients with mild, moderate, and severe renal impairment the AUC was increased by 32, 74, and 72% respectively and the profile of metabolites is changed in favor of active metabolites. Subjects with a terminal renal disease had a total exposure of 93% higher than healthy subjects.

In patients with mild and moderate hepatic impairment, the pharmacokinetics and pharmacodynamics of edoxaban are not changed. Edoxaban should be used with caution in patients with liver enzyme or bilirubin levels ≥2 and 1.5 normal values respectively [24]. Edoxaban is now marketed as coated tablets in dosages of 15 mg, 30 mg, and 60mg.

Edoxaban authorized indications are:

- Stroke and systemic embolism prevention in adult patients with non-valvular atrial fibrillation (FANV), with one or more risk factors, such as congestive heart failure, hypertension, age ≥75 years, diabetes mellitus, previous stroke or transient ischemic attack (TIA) (60 mg per day).
- Treatment of deep vein thrombosis (DVT) and pulmonary embolism (PE) and prevention of recurrences of DVT and PE in adults (60 mg per day, after the initial use of an injecting anticoagulant for at least 5 days).

In the ENGAGE AF-TIMI 48 (e.g. authorization study) both approved doses (30 mg and 60 mg) significantly reduced the risk of increased bleeding (HR 0.47 and 0.80 respectively), GI bleeding (HR 0.67 and 1.23) and the risk of death from cardiovascular causes (HR 0.85 and 0.86 respectively) compared to warfarin [25]. The relative efficacy and safety of edoxaban 60 mg compared with warfarin were independent of different clinical conditions, such as prior stroke, age, risk of falls, renal function, hepatic disease, ischemic heart disease, heart failure, valvular heart disease, or cancer [26]. After the publication of this study, a great number of substudies and post hoc analyses have been published, together with some observational studies. For example, in a study on Asian patients, edoxaban reported a reduced risk of increased bleeding and mortality for all causes compared to warfarin [27].

Edoxaban is the last NOAC to obtain marketing authorization and limited data about the effectiveness and safety of edoxaban in real-life patients are scarce but consistent with those of the pivotal clinical trial.

1.5. NOACs vs Acenocoumarol

A recent study compared the efficacy and safety of NOACs with acenocoumarol optimally anticoagulated patients. This included 1361 patients treated with acenocoumarol with 100% time in therapeutic range (TTR) and followed in follow-up for 6.5 years. Compared to an optimally anticoagulated population, dabigatran 110 mg had the highest estimated stroke reduction rate (0.97%/year vs 1.47%/year), the benefit was higher than that of the RE-LY study. Apixaban showed the highest estimated reduction (1.81%/year vs 2.83%/year) for major bleeding and the largest estimated reduction of mortality (2.68%/year). Only apixaban showed a significant reduction of gastrointestinal bleeding, compared to acenocoumarol (0.69%/year vs 1.10%/year); this reduction was significantly higher than that of the ARISTOTLE study.

All NOCAs showed significantly lower risk rates for intracranial hemorrhage and had a Net Clinical Benefit (NCB) positive compared to acenocoumarol. Apixaban showed the highest NCB estimated (2.64; 95%CI 2.34–2.96).

The authors assert that in patients with AF optimally anticoagulated with acenocoumarol if treated with NOCAs is evident the estimated risk reduction of all clinical outcomes and apixaban has the best profile of effectiveness and safety [28].

Although Real World data and meta-analyses show apixaban has a better tolerability profile, in the absence of a head-to-head trial, it may be inappropriate to define one of the NOCAs as the most appropriate treatment for stroke prevention in a patient with NVAF [29].

Table 1. ICD9-CM codes considered in the analysis.

ICD9-CM	Diagnosis
325	phlebitis and thrombophlebitis of intracranial venous sinuses
410	acute myocardial infarction
411	other acute and subacute forms of ischemic heart disease
412	previous myocardial infarction
430	subarachnoid hemorrhage
431	cerebral hemorrhage
432	other and unspecified intracranial haemorrhages
434	occlusion of cerebral arteries
435	transient cerebral ischemia
444	embolism and arterial thrombosis
453	embolism and thrombosis of other veins
531	stomach ulcer
532	duodenal ulcer
533	peptic ulcer, site not specified
534	gastrodjejunum ulcer
578	gastrointestinal hemorrhage
415.0	acute cor pulmonale
415.1	pulmonary embolism and pulmonary infarction
415.11	pulmonary embolism and iatrogenic pulmonary infarction
415.19	other forms of pulmonary embolism and pulmonary infarction
432.0	non traumatic extradural hemorrhage
432.1	subdural hemorrhage
432.9	intracranial hemorrhage not specified
434.0	cerebral thrombosis
434.00	cerebral thrombosis without mention
434.01	cerebral thrombosis with infarction
434.1	cerebral embolism
434.10	brain embolism without mention of cerebral infarction
434.11	cerebral embolism with cerebral infarction
434.9	occlusion of cerebral artery, not specified
434.90	unspecified cerebral artery occlusion without mention of cerebral infarction
434.91	unspecified cerebral artery occlusion with cerebral infarction
435.8	other specified transient cerebral ischemia
435.9	unspecified transient cerebral ischemia
437.1	other generalized cerebral ischemic vasculopathies
444.0	abdominal aorta embolism and thrombosis
444.1	thoracic aorta embolism and thrombosis
444.21	embolism and thrombosis of the arteries of the upper limbs
444.22	embolism and thrombosis of the arteries of the lower limbs
444.8	embolism and thrombosis of other specified arteries
444.9	unspecified artery embolism and thrombosis
451.0	phlebitis and thrombophlebitis of the superficial vessels of the lower extremities
451.11	phlebitis and thrombophlebitis of the femoral vein
451.19	phlebitis and thrombophlebitis of other veins
451.2	phlebitis and thrombophlebitis of the lower extremities, not specified
451.81	phlebitis and thrombophlebitis of the iliac vein
451.82	phlebitis and thrombophlebitis of the superficial veins of the upper extremities
451.83	phlebitis and thrombophlebitis of the deep veins of the upper extremities
451.84	unspecified phlebitis and thrombophlebitis of the upper extremities
451.89	phlebitis and thrombophlebitis of other sites
451.9	phlebitis and thrombophlebitis of unspecified sites
452	portal vein thrombosis
453.1	migrant thrombophlebitis
453.2	vena cava embolism and thrombosis
453.3	renal vein embolism and thrombosis
453.40	venous embolism and thrombosis of unspecified deep vessels of the lower limbs
453.41	venous embolism and thrombosis of the deep vessels of the proximal parts of the lower limbs
453.42	venous embolism and thrombosis of the deep vessels of the distal parts of the lower limbs

453.8	embolism and thrombosis of other specified veins
453.9	embolism and thrombosis of unspecified site
569.3	rectum and anus hemorrhage
578.0	hematemesis
578.1	melaena and rettorragia
578.9	gastrointestinal hemorrhage, not specified
593.81	kidney vascular diseases
596.7	intramural bladder hemorrhage

Table 2. Aggregated Discharge Diagnosis 2017 and Related Incidence with Confidence Interval (CI)—patients treated with NOACs.

ICD Diagnosis of Discharge 2017	Number of Cases	Incidence/100 Persons per Year	CI 95% −	CI 95% +
Myocardial infarction	63	4.17	3.91	4.44
Brain hemorrhage	4	0.27	0.20	0.33
Brain thrombosis and embolism	52	3.45	3.20	3.69
Ischemic events	14	0.93	0.80	1.05
Embolism and vascular thrombosis	24	1,.59	1.43	1.75
GI Hemorrhage	11	0.73	0.62	0.84
Pulmonary embolism	29	1.92	1.74	2.10

Table 3. Aggregated Discharge Diagnosis 2018 and Related Incidence with Confidence Interval (CI)—patients treated with NOACs.

ICD Diagnosis of Discharge 2018	Number of Cases	Incidence/100 Persons per Year	CI 95% −	CI 95% +
Myocardial infarction	15	1.19	0.58	1.8
Brain hemorrhage	6	0.47	0.09	0.85
Brain thrombosis and embolism	10	0.79	0.3	1.28
Ischemic events	4	0.32	0.01	0.63
Embolism and vascular thrombosis	1	0.08	(−0.07)	0.23
GI Hemorrhage	7	0.55	0.14	0.96
Pulmonary embolism	2	0.16	(−0.06)	0.38

Table 4. Aggregated Discharge Diagnosis 2017 And Related Incidence with Confidence Interval (CI)—patients treated with VKAs.

ICD Diagnosis of Discharge 2017 VKAs	Number of Cases	Incidence/100 Persons per Year	CI 95% −	CI 95% +
Myocardial infarction	65	1.73	1.66	1.80
Bain hemorrhage	12	0.32	0.29	0.35
Brain thrombosis and embolism	30	0.80	0.75	0.84
Ischemic events	17	0.45	0.42	0.49
Embolism and vascular thrombosis	23	0.61	0.57	0.65
GI Hemorrhage	36	0.96	0.91	1.01
Pulmonary embolism	11	0.29	0.26	0.32

Table 5. Aggregated Discharge Diagnosis 2018 and Related Incidence with Confidence Interval (CI)—patients treated with VKAs.

ICD Diagnosis of Discharge 2018 VKAs	Number of cases	Incidence/100 Persons per Year	CI 95% −	CI 95% +
Myocardial infarction	42	1.31	0.91	1.71
Brain hemorrhage	24	0.75	045	1.05
Brain thrombosis and embolism	30	0.94	0.6	1.28
Ischemic events	10	0.31	0.12	0.5
Embolism and vascular thrombosis	16	0.5	0.25	0.75
GI Hemorrhage	28	0.88	0.56	1.2
Pulmonary embolism	8	0.25	0.08	0.42

References

1. Hauel, N.H.; Nar, H.; Priepke, H.; Ries, U.; Stassen, J.-M.; Wienen, W. Structure-Based Design of Novel Potent Nonpeptide Thrombin Inhibitors. *J. Med. Chem.* **2002**, *45*, 1757–1766, doi:10.1021/jm0109513.
2. Stangier, J.; Stähle, H.; Rathgen, K.; Fuhr, R. Pharmacokinetics and Pharmacodynamics of the Direct Oral Thrombin Inhibitor Dabigatran in Healthy Elderly Subjects. *Clin. Pharmacokinet.* **2008**, *47*, 47–59, doi:10.2165/00003088-200847010-00005.
3. Heidbuchel, H.; Verhamme, P.; Alings, M.; Antz, M.; Hacke, W.; Oldgren, J.; Sinnaeve, P.; Camm, A.J.; Kirchhof, P. European Heart Rhythm Association Practical Guide on the use of new oral anticoagulants in patients with non-valvular atrial fibrillation. *EP Eur.* **2013**, *15*, 625–651, doi:10.1093/europace/eut083.
4. Cabral, K.P. Pharmacology of the new target-specific oral anticoagulants. *J. Thromb. Thrombolysis* **2013**, *36*, 133–140, doi:10.1007/s11239-013-0929-5.
5. *Riassunto delle Caratteristiche del Prodotto, Pradaxa*; Boehringer Ingelheim International GmbH: Ingelheim am Rhein, Germany, 2019.
6. Connolly, S.J.; Ezekowitz, M.D.; Yusuf, S.; Eikelboom, J.; Oldgren, J.; Parekh, A.; Pogue, J.; Reilly, P.A.; Themeles, E.; Varrone, J.; et al. Dabigatran versus Warfarin in Patients with Atrial Fibrillation. *N. Engl. J. Med.* **2009**, *361*, 1139–1151, doi:10.1056/NEJMoa0905561.
7. Artang, R.; Rome, E.; Nielsen, J.D.; Vidaillet, H.J. Meta-Analysis of Randomized Controlled Trials on Risk of Myocardial Infarction from the Use of Oral Direct Thrombin Inhibitors. *Am. J. Cardiol.* **2013**, *112*, 1973–1979, doi:10.1016/j.amjcard.2013.08.027.
8. Lee, C.J.-Y.; Gerds, T.A.; Carlson, N.; Bonde, A.N.; Gislason, G.H.; Lamberts, M.; Olesen, J.B.; Pallisgaard, J.L.; Hansen, M.L.; Torp-Pedersen, C. Risk of Myocardial Infarction in Anticoagulated Patients With Atrial Fibrillation. *J. Am. Coll. Cardiol.* **2018**, *72*, 17–26, doi:10.1016/j.jacc.2018.04.036.
9. Ntaios, G.; Papavasileiou, V.; Makaritsis, K.; Vemmos, K.; Michel, P.; Lip, G.Y.H. Real-World Setting Comparison of Nonvitamin-K Antagonist Oral Anticoagulants Versus Vitamin-K Antagonists for Stroke Prevention in Atrial Fibrillation. *Stroke* **2017**, *48*, 2494–2503, doi:10.1161/STROKEAHA.117.017549.
10. Kreutz, R. Pharmacodynamic and pharmacokinetic basics of rivaroxaban. *Fundam. Clin. Pharmacol.* **2012**, *26*, 27–32, doi:10.1111/j.1472-8206.2011.00981.x.
11. *Riassunto delle Caratteristiche del Prodotto, Xarelto*; Bayer: Leverkusen, Germany, 2019.
12. Mueck, W.; Lensing, A.W.A.; Agnelli, G.; Decousus, H.; Prandoni, P.; Misselwitz, F. Rivaroxaban. *Clin. Pharmacokinet.* **2011**, *50*, 675–686, doi:10.2165/11595320-000000000-00000.
13. Patel, M.R.; Mahaffey, K.W.; Garg, J.; Pan, G.; Singer, D.E.; Hacke, W.; Breithardt, G.; Halperin, J.L.; Hankey, G.J.; Piccini, J.P.; et al. Rivaroxaban versus Warfarin in Nonvalvular Atrial Fibrillation. *N. Engl. J. Med.* **2011**, *365*, 883–891, doi:10.1056/NEJMoa1009638.
14. Hernandez, I.; Zhang, Y. Comparing Stroke and Bleeding with Rivaroxaban and Dabigatran in Atrial Fibrillation: Analysis of the US Medicare Part D Data. *Am. J. Cardiovasc. Drugs* **2017**, *17*, 37–47, doi:10.1007/s40256-016-0189-9.
15. Frost, C.; Wang, J.; Nepal, S.; Schuster, A.; Barrett, Y.C.; Mosqueda-Garcia, R.; Reeves, R.A.; LaCreta, F. Apixaban, an oral, direct factor Xa inhibitor: Single dose safety, pharmacokinetics, pharmacodynamics and food effect in healthy subjects. *Br. J. Clin. Pharmacol.* **2013**, *75*, 476–487, doi:10.1111/j.1365-2125.2012.04369.x.
16. Wong, P.C.; Crain, E.J.; Xin, B.; Wexler, R.R.; Lam, P.Y.S.; Pinto, D.J.; Luettgen, J.M.; Knabb, R.M. Apixaban, an oral, direct and highly selective factor Xa inhibitor: In vitro, antithrombotic and antihemostatic studies. *J. Thromb. Haemost.* **2008**, *6*, 820–829, doi:10.1111/j.1538-7836.2008.02939.x.
17. Carreiro, J.; Ansell, J. Apixaban, an oral direct Factor Xa inhibitor: Awaiting the verdict. *Expert Opin. Investig. Drugs* **2008**, *17*, 1937–1945, doi:10.1517/13543780802528625.
18. *Riassunto delle Caratteristiche del Prodotto, Eliquis*; Bristol-Myers Squibb, S.r.l.: Rome, Italy, 2019.
19. Connolly, S.J.; Eikelboom, J.; Joyner, C.; Diener, H.C.; Hart, R.; Golitsyn, S.; Flaker, G.; Avezum, A.; Hohnloser, S.H.; Diaz, R.; et al. Apixaban in patients with atrial fibrillation. *N. Engl. J. Med.* **2011**, doi:10.1056/NEJMoa1007432.

20. Lopes, R.D.; Alexander, J.H.; Al-Khatib, S.M.; Ansell, J.; Diaz, R.; Easton, J.D.; Gersh, B.J.; Granger, C.B.; Hanna, M.; Horowitz, J.; et al. Apixaban for Reduction In Stroke and Other ThromboemboLic Events in Atrial Fibrillation (ARISTOTLE) trial: Design and rationale. *Am. Heart J.* **2010**, *159*, 331–339, doi:10.1016/j.ahj.2009.07.035.
21. Lip, G.Y.H.; Keshishian, A.; Li, X.; Hamilton, M.; Masseria, C.; Gupta, K.; Luo, X.; Mardekian, J.; Friend, K.; Nadkarni, A.; et al. Effectiveness and Safety of Oral Anticoagulants Among Nonvalvular Atrial Fibrillation Patients. *Stroke* **2018**, *49*, 2933–2944, doi:10.1161/STROKEAHA.118.020232.
22. Tepper, P.; Mardekian, J.; Masseria, C.; Phatak, H.; Kamble, S.; Abdulsattar, Y.; Petkun, W.; Lip, G.Y.H. Real-world comparison of bleeding risks among non-valvular atrial fibrillation patients on apixaban, dabigatran, rivaroxaban: Cohorts comprising new initiators and/or switchers from warfarin. *Eur. Heart J.* **2015**, *36*, 339.
23. Deitelzweig, S.; Bruno, A.; Tate, N.; Ogbonnaya, A.; Shah, M.; Farrelly, E.; Lokhandwala, T.; Gupta, K.; Eaddy, M. Major bleeding, hospitalisation rates and healthcare costs among non-valvular atrial fibrillation patients naive to oral anticoagulation and newly treated with novel oral anticoagulants. *Eur. Heart J.* **2016**, *36*, 338.
24. *Riassunto delle Caratteristiche del Prodotto, Lixiana*; Daiichi Sankyo Europe GmbH: Rome, Italy, 2019; pp. 1–33.
25. Giugliano, R.P.; Ruff, C.T.; Braunwald, E.; Murphy, S.A.; Wiviott, S.D.; Halperin, J.L.; Waldo, A.L.; Ezekowitz, M.D.; Weitz, J.I.; Špinar, J.; et al. Edoxaban versus Warfarin in Patients with Atrial Fibrillation. *N. Engl. J. Med.* **2013**, *369*, 2093–2104, doi:10.1056/NEJMoa1310907.
26. Cervantes, C.E.; Merino, J.L.; Barrios, V. Edoxaban for the prevention of stroke in patients with atrial fibrillation. *Expert Rev. Cardiovasc. Ther.* **2019**, *17*, 319–330, doi:10.1080/14779072.2019.1598263.
27. Lee, S.-R.; Choi, E.-K.; Han, K.-D.; Jung, J.-H.; Oh, S.; Lip, G.Y.H. Edoxaban in Asian Patients With Atrial Fibrillation. *J. Am. Coll. Cardiol.* **2018**, *72*, 838–853, doi:10.1016/j.jacc.2018.05.066.
28. Esteve-Pastor, M.A.; Rivera-Caravaca, J.M.; Roldán, V.; Orenes-Piñero, E.; Romiti, G.F.; Romanazzi, I.; Bai, Y.; Carmo, J.; Proietti, M.; Marín, F.; et al. Estimated Effectiveness and Safety of Nonvitamin K Antagonist Oral Anticoagulants Compared With Optimally Acenocoumarol Anticoagulated "Real-World" in Patients With Atrial Fibrillation. *Am. J. Cardiol.* **2018**, *122*, 785–792, doi:10.1016/j.amjcard.2018.05.012.
29. Camm, A.J.; Lip, G.Y.H.; De Caterina, R.; Savelieva, I.; Atar, D.; Hohnloser, S.H.; Hindricks, G.; Kirchhof, P.; Bax, J.J.; Baumgartner, H.; et al. 2012 focused update of the ESC Guidelines for the management of atrial fibrillation. *Eur. Heart J.* **2012**, *33*, 2719–2747, doi:10.1093/eurheartj/ehs253.
